# Mechanical Circulatory Assist Devices: Which Is the Best Device as Bridge to Heart Transplantation?

**DOI:** 10.21470/1678-9741-2021-0562

**Published:** 2022

**Authors:** Adriana Natucci Hette, Marcelo Luiz Peixoto Sobral

**Affiliations:** 1 Faculdade de Medicina, Centro Universitário Faculdade das Américas, São Paulo, São Paulo, Brazil; 2 Hospital Beneficência Portuguesa de São Paulo, São Paulo, São Paulo, Brazil

**Keywords:** Heart Transplantation, Heart Failure, Extracorporeal Membrane Oxygenation

## Abstract

**Introduction:**

Heart transplantation is the recommended treatment method for patients with advanced heart failure that is refractory to clinical treatment. Due to the progressive severity of these patients and the impossibility of performing the transplant in a short term, there are mechanical circulatory assist devices that can offer necessary hemodynamic support and clinical stability in the period preceding the heart transplant surgery. The present study aims to address and describe the main devices used as bridges for heart transplantation, as well as to analyze their advantages and disadvantages.

**Methods:**

This work is a literature review, developed with scientific production in the period from 2010 to 2020, that focus on circulatory assist devices as a bridge for heart transplantation.

**Results:**

These devices are characterized as a bridge for transplantation. Short-term or temporary devices are those used for hemodynamic support to stabilize the individual clinically in the presence of refractory cardiogenic shock. And long-term devices are indicated for stable patients with long-term strategic planning.

**Conclusion:**

According to the present study, it is possible to observe that there is a wide variety of devices available on the market, enabling the most appropriate choice according to the patient’s need.

**Table:** 

Abbreviations, Acronyms & Symbols	
ECMO	= Extracorporeal membrane oxygenation
HF	= Heart failure
IAB	= Intra-aortic balloon
INTERMACS	= Interagency Registry for Mechanically Assisted Circulatory Support
IVC	= Inferior vena cava
LV	= Left ventricle
LVEF	= Left ventricular ejection fraction
MCAD	= Mechanical circulatory assist device
OHT	= Orthotopic heart transplantation
PA	= Pulmonary artery
PH	= Pulmonary hypertension
PI	= Pulse index
RV	= Right ventricle
SVC	= Superior vena cava

## INTRODUCTION

Heart failure (HF) is considered the main cause of cardiovascular hospitalization in Brazil and is a complex clinical syndrome, in which the heart is unable to pump blood to supply the metabolic tissue demand or only in the face of high filling pressure. This condition can be caused by structural or functional cardiac changes and is characterized by typical signs and symptoms, which result from reduced cardiac output and/or high filling pressures at rest or on exertion^[1]^. It is estimated that about 1-2% of the population have HF, and approximately half of these individuals have reduced ejection fraction^[2]^.

HF may be due to an abnormality in systolic function, producing a reduction in stroke volume (systolic HF), or an abnormality in diastolic function, leading to a defect in ventricular filling (diastolic HF), determining typical and characteristic symptoms in each type of failure. Also, HF can be classified according to the ejection fraction (preserved, intermediate, and reduced), the severity of symptoms (functional classification of the New York Heart Association [or NYHA]), and the time and progression of the disease (different stages)^[1]^.

The main classification for HF is based on the left ventricular ejection fraction (LVEF) - normal LVEF (≥ 50%), heart failure with preserved ejection fraction (or HFpEF), and reduced LVEF (< 40%), heart failure with reduced ejection fraction (or HFrEF). In addition to these two classifications, there are patients with an ejection fraction between 40 and 49% who recently came to be defined as heart failure with intermediate ejection fraction (or HFiEF) or mid-range (or HFmrEF)^[3]^.

In the last three decades, there has been a great evolution in the treatment of chronic HF, however, there is still an important limitation in the quality of life of these patients - a significant part of these patients develop refractoriness to classical treatment and hospitalizations with death and readmission rates in the six-month period around 50%^[2]^.

Heart transplantation is the recommended treatment method for patients with advanced HF and refractory to conservative treatment^[4,5]^. However, the destination of the organ for transplantation implies ethical issues and scarce resources, prioritizing the individuals most likely to survive in the long term^[6]^. Although heart transplantation is the recommended treatment in these cases of HF, it is a limited procedure, mainly due to the number of donors available and the recipient’s contraindications, such as pulmonary hypertension (PH) secondary to HF^[7]^.

The main indications for heart transplantation, according to the third Brazilian cardiac transplantation guideline^[7]^, are: advanced HF and peak VO2 = 12 ml/kg/minute in patients using beta-blockers (recommendation I, level of evidence B); advanced HF and peak VO2 = 14 ml/kg/minute in patients intolerant to beta-blockers (recommendation I, level of evidence B); advanced HF in dependence on inotropic drugs and/or mechanical circulatory support (recommendation I, level of evidence C); advanced HF functional class III persistent and IV with optimized treatment in the presence of other factors of poor prognosis (recommendation I, level of evidence C); symptomatic ventricular arrhythmias that are refractory to management with drugs, electrical devices, and ablation procedures (recommendation I, level of evidence C).

The prognostic evaluation of the patient in the heart transplantation queue can be done using the Interagency Registry for Mechanically Assisted Circulatory Support (INTERMACS) classification. Although it was not created to define criteria for the surgery, it is useful in clinical and prognostic evaluations, when indicating therapies for advanced HF and/or cardiogenic shock^[8,9]^. It is divided into seven categories, with patients in critical condition (INTERMACS 1 and 2) configuring situations in which the perioperative risk for transplantation is very unfavorable^[10]^.

Due to the progressive severity of the patients and the impossibility of performing the transplant in a short term, there are devices that can offer hemodynamic support and clinical stability, necessary in the period preceding the heart transplant surgery. These devices are characterized as a bridge for transplantation^[11]^. Mechanical circulatory assist devices (MCAD) can be classified in several ways, such as length of stay (short or long), type of implantation technique (paracorporeal or fully implantable), and type of flow (pulsatile or continuous)^[12]^. Short-term or temporary devices are those used for hemodynamic rescue in order to stabilize the individual clinically in the presence of refractory cardiogenic shock. And long-term devices are indicated for stable patients with long-term strategic planning. In Brazil, there are currently three long-term devices available: HeartMate II®, Berlin Heart INCOR®, and HeartWare®^[7]^.

The number of patients supported by long-term MCAD, especially as a bridge for transplantation, is increasing because these individuals are progressively improving their survival.

However, there is a restriction regarding the prioritization of the use of MCAD as a bridge for transplantation, and it should be directed to those individuals with complications, in the impossibility of changing the device: clinical deterioration despite the device, intractable infection related to the device, mechanical dysfunction of the device, thromboembolic events, device thrombosis with hemodynamic impairment, and recurrent ventricular arrhythmias^[13]^.

### Objective

This study aims to address the main devices used as a bridge for heart transplantation, as well as to analyze their indications, complications, advantages, and disadvantages and answer the following question, which is the best device to be used as a bridge for transplantation?

## METHODS

This is a descriptive literature review study, developed with scientific production from 2010 to 2020 indexed in the electronic databases Latin American and Caribbean Health Sciences Literature (or LILACS), PubMed®, and Scientific Electronic Library Online (or SciELO), which focus on circulatory assist devices as a bridge for heart transplantation. The systematic review answers a specific question and uses explicit and systematic methods to identify, select, and critically evaluate the studies, to collect and analyze the data of those studies to be included in the review.

## RESULTS

### Use of Mechanical Circulatory Support

Currently, mechanical circulatory support systems are used in three situations, according to the guidelines of the European Society of Cardiology (or ESC) and the Mechanical Circulatory Assistance Directive of the Brazilian Society of Cardiology: a *bridge for decision*, is used in patients with clinical conditions that contraindicate heart transplantation, however, if modified, allow the patient to become a candidate for transplantation (*e.g.*, PH and neoplasms with a potential cure); *bridge for transplantation*, in this case the device can offer hemodynamic support and clinical stability until the surgery is performed, in the context of the patient’s progressive severity and the unavailability of the transplant in a short period; and *destination therapy*, when the device provides hemodynamic support and clinical stability in a patient with refractory HF, which has a contraindication for heart transplantation, thus enabling greater survival and better quality of life when compared to medical drug treatment^[11,12]^.

### Mechanical Circulatory Assist Devices

Temporary or short-term MCAD (Table 1) are commonly used for the clinical and hemodynamic stability of the patient, including the possibility of recovering cardiac function, as well as performing the transplant^[11]^. Traditionally, temporary MCAD are preferentially indicated in INTERMACS 1 and 2 patients, however those patients classified as INTERMACS 3, who are dependent on high inotropic doses or at high risk of hemodynamic instability, can be considered candidates^[11,14]^.

**Table 1:** Main short-stay devices used in Brazil.

Device	Mechanism	Access way	Hemodynamic support
Intra-aortic balloon	Pneumatic	Percutaneous	0,5 L/min
Extracorporeal membrane oxygenation	Centrifugal	Percutaneous/direct by thoracotomy	> 4,5 L/min
TandemHeart™	Centrifugal	Percutaneous	4 L/min
Impella®	Axial	Percutaneous or dissection	2,5-5 L/min
CentriMag®	Centrifugal	Direct by thoracotomy	Up to 8-10 L/min
EXCOR®	Pulsatile	Direct by thoracotomy	Up to 8 L/min

In order to assist heart transplantation candidates who may require the benefits of circulatory assistance, devices have been developed that will function for long periods until a donor is obtained (Table 2). During this period, MCAD must offer adequate blood flow with the least degree of damage to blood elements, in addition to a lower rate of activation of the different cascade systems, such as the coagulation cascade, and allow the patient to walk^[15]^.

**Table 2:** Classification of long-term mechanical circulatory assist devices.

Generation	Device
First	HeartMate I® (HeartMate XVE), Novacor®, LionHeart LVAD 2000®
Second	MicroMed DeBakey®, Jarvik 2000®, HeartMate II®
Third	HeartMate III®, INCOR®, VentrAssist®, Levacor®, Terumo DuraHeart®, CorAide®, Heartware®

The first generation of MCAD used pulsatile flow mechanisms through pneumatic propulsion. The implantation was done in an infradiaphragmatic position^[16]^. The main disadvantages were its size, the noise produced, the risk of infection and embolization, and its mechanical durability, estimated in just two years^[17]^.

The second generation of MCAD uses axial continuous flow systems. The blood is pumped through an impeller, with a small high-speed system, so there is no need for valves. With this evolution, there was a decrease in the size and weight of the devices, noise reduction, and implantation surgery involving a more limited area, which enables implantation in patients with a smaller body area^[18]^.

The HeartMate III® is the latest third-generation long-life device, it is a centrifugal MCAD with an all-magnetic impeller. MOMENTUM 3 (the Multicenter Study of MagLev Technology in Patients Undergoing Mechanical Circulatory Support Therapy with HeartMate III®) compared HeartMate III® with HeartMate II® and obtained promising follow-up data^[12]^.

### Devices Available in Brazil

#### Short Stay

1) The intra-aortic balloon (IAB), which has an aortic counterpulsation mechanism, increases the diastolic pressure at the root of the aorta, thereby causing an increase in coronary perfusion, reducing afterload with a consequent increase in output around 15%. IAB is usually inserted by puncture of the femoral artery and positioned in the descending thoracic aorta, immediately distal to the origin of the left subclavian artery^[11]^.

2) Extracorporeal membrane oxygenation (ECMO) is a temporary invasive mechanical support that provides partial or total cardiopulmonary support for patients in cardiogenic shock and/or acute respiratory failure. It can be of two types: venous-arterial and venous-venous. It is a device with quick installation, applicable to most patients, and which reverses circulatory failure and/or anoxia quickly. The implant is done through percutaneous cannulation or direct by thoracotomy. In venous-venous ECMO, both drainage and blood infusion are made exclusively from the venous system; venous-arterial ECMO is a temporary mechanical support (one to 30 days) for patients with potential functional recovery or as a bridge for decision, transplantation, or long-term MCAD^[11]^.

3) TandemHeart™ is a device that pumps blood from the left atrium through a cannula inserted transseptally by an extracorporeal centrifugal pump into the arterial iliofemoral system. In this case, both the TandemHeart™ and the left ventricle (LV) are responsible for sending the flow to the aorta, that is, they work in parallel. TandemHeart™ consists of a transseptal cannula, centrifugal pump, femoral arterial cannula, and console^[11]^.

Cannulation of the femoral vein is performed with a 21 F introducer for transseptal puncture, and the cannula is positioned in the left atrium. Then, the cannulation of the femoral artery is performed with a 15 or 17 F catheter. The 15 F cannulas produce a flow rate of 3.5 L/min and the 17 F cannula produces 5 L/min. Full anticoagulation of the patient is required, and the length of stay with the device is up to 30 days. It is important to note that even after the device is removed, the patient remains with residual atrial septal defect^[11]^.

**Fig. 1:**
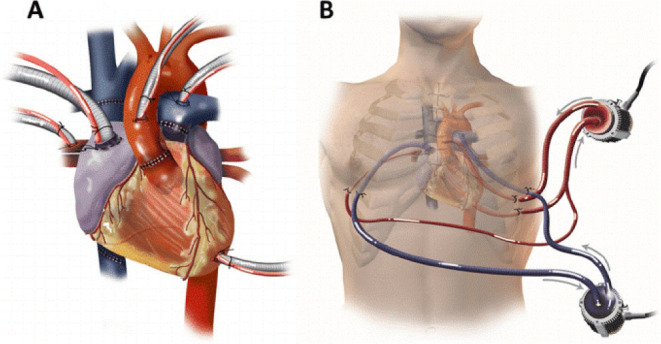
CentriMag®. A) Cannulation strategy: a CentriMag® was used to support the left heart with cannulation via the left atrium, left ventricle, and aorta. Another CentriMag® was used to support the right ventricle with cannulation via the right atrium and pulmonary artery. This strategy allowed for excellent flows from both devices and complete decompression of the heart. B) CentriMag® access strategy: all cannulas were removed from the chest through intercostal or subcostal incisions, allowing sternotomy closure. Kaczorowski et al. Journal of Cardiothoracic Surgery, 2013.

**Fig. 2:**
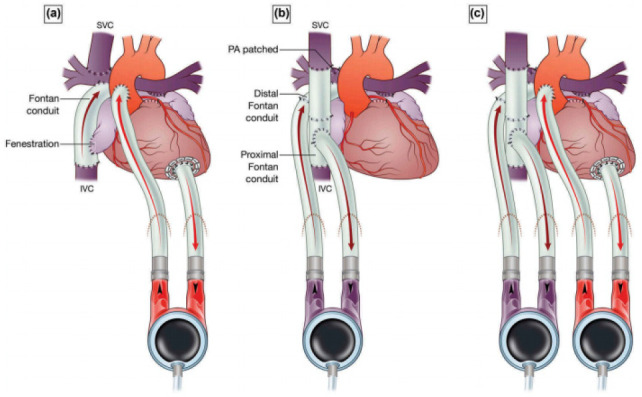
Berlin Heart EXCOR® configurations. A) Supports systemic circulation; B) supports pulmonary circulation; and C) supports both circulations. IVC=inferior vena cava; PA=pulmonary artery; SVC=superior vena cava. Courtesy of Professor Igor E. Konstantinov, reproduced from E. Buratto et al. Expert Review of Medical Devices, 2017.

4) Impella® consists of a continuous axial flow pump, which draws blood from the LV into the aorta, that is, in this case, it works in series with the LV. There are three types of Impella® on the market today, the difference between them is the flow allowed - flow of 2.5 L/min (Impella® 2.5), 4 L/min (Impella® CP), or 5.0 L/min (Impella® 5.0). In Brazil, currently, the available model is the Impella® CP. Cannulation of the femoral artery is performed for the Impella® implant, followed by the retrograde passage of the device through the aortic valve and the positioning of the microaxial pump in the ascending aorta by fluoroscopy. Full patient anticoagulation is required. The length of stay with the device is five to seven days^[11]^.

5) CentriMag® (Figure 1) is a continuous flow centrifugal pump that uses magnetic levitation for rotation. It provides a flow of up to 10 L/min with low shear stress, minimizing thrombogenicity and allowing moderate levels of anticoagulation and minimal hemolysis during support. CentriMag® can be single or biventricular and requires a median sternotomy for its installation. Its implant uses simple and direct cannulation, including no extracorporeal circulation type right atrium (RA) to the pulmonary artery trunk (right support), and atrium or LV to the ascending aorta (left support). CentriMag® is authorized in the United States of America for support for up to 30 days, although there are reports of use for up to three months without failure of the pump or an increase in thromboembolic complications^[11]^.

6) Berlin Heart EXCOR® (Figure 2) is a pulsatile flow pump that supplies up to 8 L/min, with batteries attached to a transport system, which allows walking for up to 10 hours. As well as CentriMag®, EXCOR® can offer single or biventricular support, it is implanted through median thoracotomy, and requires specific cannulas for its cannulation. Although it is a paracorporeal device, it has greater durability than CentriMag®, for example, and can remain for months as hemodynamic support in patients with cardiogenic shock. In the United States of America, the EXCOR® pediatric model is considered a long-term device^[11]^.

#### Long Stay

**Fig. 3:**
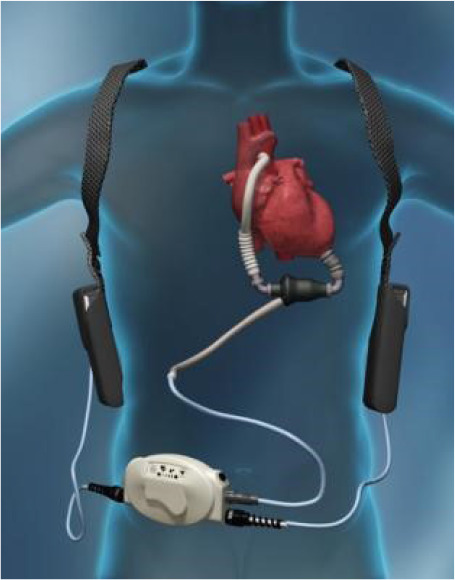
HeartMate II® (Thoratec Corporation).

1) HeartMate II® (Figure 3) is a continuous flow device and is part of the second generation of MCAD. When compared to the pulsatile device, it showed improvement in stroke-free survival or reoperation in two years (46% *vs.* 11% pulsatile), in overall survival (58% *vs.* 24% in two years), functional capacity, and quality of life^[19]^. HeartMate II® can be used both as a bridge for transplantation and as destination therapy for those patients who have a contraindication to transplantation. The parameters of this device are pump flow (in L/min), pump speed (in rpm), pulse index (PI), and pump power (in watts). The main parameter to regulate it is the pump speed. If the speed is very high, the LV is low in volume, which can lead to deviated septum and hemodynamic collapse (a drop in PI is observed, which represents how much the native heart helps in contraction). The power of the pump represents the energy needed to run the pump; if too high, it may suggest obstruction or thrombus. Patients with this implanted device should be kept under standard treatment for HF and anticoagulation, if without contraindications.

The main complications of this device are stroke or embolic events, bleeding, infection, arrhythmias, and hemodynamic collapse due to deviated interventricular septum. However, the main cause of morbidity and mortality in these patients is dysfunction of the right ventricle (RV), since this is a left assist device only^[19]^.

**Fig. 4:**
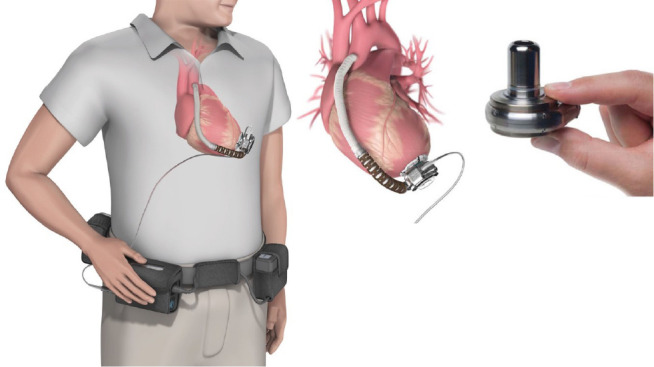
HeartWare® (HeartWare Inc, Framingham, Massachusetts, United States of America).

**Fig. 5:**
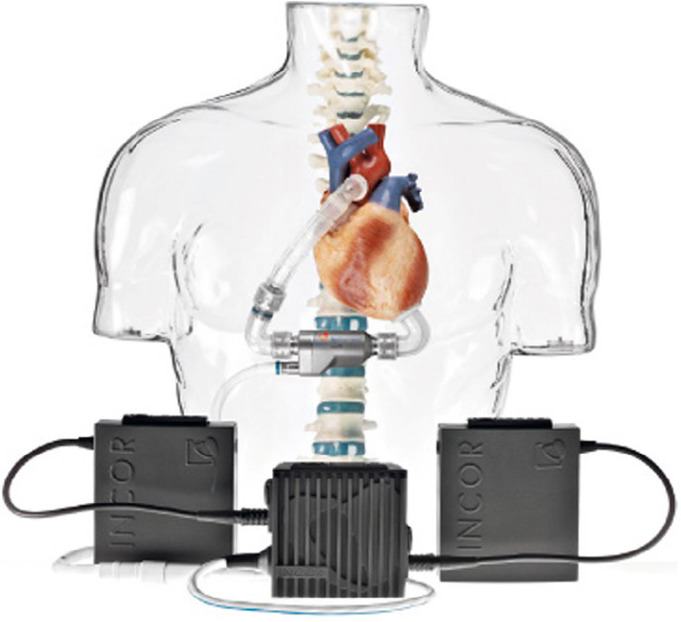
Berlin Heart INCOR® (Berlin Heart, www.berlinheart.com).

2) HeartWare® (Figure 4) - Ventricular Assist System (HeartWare Inc, Framingham, Massachusetts, United States of America) is an implantable device with a continuous flow centrifugal blood pumping function and is part of the third generation of MCAD. Among the advantages, when compared to other commercial ventricular assist device (or VAD) pumps, HeartWare® ventricular assist device (or HVAD) is integrated into the flow cannula, allowing implantation in the pericardial space, and does not require abdominal surgery to form a pocket. HeartWare® has a controller that allows the maintenance of a record so that an analysis of the power and flow of the device can be made, thus facilitating its adaptation to the needs of the patient^[20]^.

3) Berlin Heart INCOR® (Figure 5), as well as the HeartWare®, is a very small pump, which also facilitates its use in the pediatric population, composed of cannulas for entry and exit into the LV and ascending aorta, respectively. There is only one transcutaneous cannula that protrudes from the patient’s body. The pump and all the cannulas that connect to the heart remain inside the body cavity^[18]^.

**Fig. 6:**
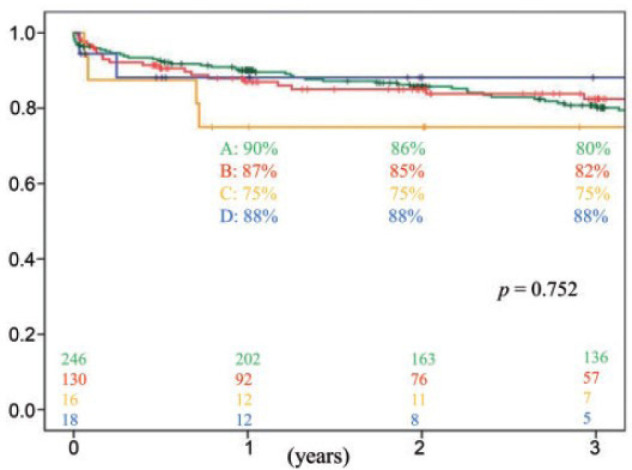
Overall survival rates after OHT in each group. Group A, heart transplantation without mechanical circulatory support (green); Group B, long-standing MCAD bridge (red); Group C, bridge with short-term MCAD (yellow); and Group D, bridge with short- and long-term devices (blue). Yoshioka D, Li B, Takayama H, Garan RA, Topkara VK, Han J et al. Outcome of heart transplantation after bridge-to-transplant strategy using various mechanical circulatory support devices. Interact CardioVasc Thorac Surg. 2017;25:918-24.MCAD=mechanical circulatory assist device; OHT=orthotopic heart transplantation.

According to a study carried out in 2017 by the Department of Surgery at Columbia University in New York, the number of patients with long-term devices as a bridge to transplantation, as well as the duration of their use, has been increasing in large proportion, as has the rate of survival (Figure 6)^[21]^.

## DISCUSSION

Even within the short-stay category, some devices allow their use for a longer period, as is the cases with ECMO and EXCOR® and, in some cases, with CentriMag®, which make them an appropriate choice for those individuals who will remain waiting for a transplant for a long time. Other advantages of these three devices are a greater hemodynamic support, allowing large blood flows. In addition, CentriMag® has less thrombogenicity when compared to other short-stay devices. The disadvantages in relation to these three MCAD is the need for thoracotomy for their implantation, in the case of ECMO there is the option of being done percutaneously.

IAB has the advantage of being percutaneously implanted, however it has a restricted flow of 0.5 L/min, and the permanence with the device is up to 72 hours; this MCAD is reserved for acute cases that evolve with serious complications, such as cardiogenic shock, while TandemHeart™ and Impella® offer greater hemodynamic support (up to 4 L/min and to 5 L/min, respectively), both of which are inserted percutaneously. The length of stay with Impella® is very short, up to seven days, since the TandemHeart™ allows its use for up to 30 days, requiring full patient anticoagulation in both cases, which can be a disadvantage. Another disadvantage of TandemHeart™ is the residual interatrial communication after its removal.

In the case of long-term MCAD, only three models are available for use in Brazil. The HeartMate II®, being a second-generation device, has no valves and provides a continuous flow with an axial mechanism, it is regulated through the flow speed, which should not be kept too high, due to the risk of thrombus formation. In addition, its assistance is exclusive to LV, for this reason its biggest complication that also becomes a disadvantage is the dysfunction of the RV. HeartWare® and INCOR® are third-generation devices, also offering a continuous flow, but with a centrifugal mechanism. The advantage of HeartWare® is the flow recording, which allows greater control over the device because it can be adapted to the patient’s needs. INCOR®, being a small pump, is widely used in pediatric patients, in addition to the fact that it is easily implanted in the patient and has only one cannula that is externalized from the individual.

## CONCLUSION

According to the present study, it is possible to observe that there is a wide variety of devices available on the market, enabling the most appropriate choice according to the patient’s need. Short-stay devices are still the most used as a bridge for transplantation, while long-stay devices are preferred as a bridge for decision and/or destination therapy.

From the graphical analysis of the use of the devices, it is possible to verify that even though they are less used as a bridge for transplantation compared to short-term devices, long-term devices have a better long-term result and a higher post-transplant survival rate. Among long-term devices, HeartWare® proved to be the best option, as it is a third-generation device and allows for flow recording, and therefore offers greater control and adaptation to the user’s needs.
